# Survival Prognosis, Tumor Immune Landscape, and Immune Responses of ADAMTS14 in Clear Cell Renal Cell Carcinoma and Its Potential Mechanisms

**DOI:** 10.3389/fimmu.2022.790608

**Published:** 2022-04-29

**Authors:** Yinhao Chen, Hao Ji, Shouyong Liu, Qianwei Xing, Bingye Zhu, Yi Wang

**Affiliations:** ^1^ Department of Urology, Affiliated Hospital of Nantong University, Nantong, China; ^2^ Department of Urology, Tumor Hospital Affiliated to Nantong University, Nantong, China; ^3^ Department of Urology, Affiliated Nantong Hospital of Shanghai University (The Sixth People’s Hospital of Nantong), Nantong, China; ^4^ Department of Urology, The First Affiliated Hospital of Nanjing Medical University, Nanjing, China

**Keywords:** ADAMTS14, prognosis, immunity, immune responses, clear cell renal cell carcinoma

## Abstract

**Background:**

ADAMTS14 played a crucial role in the formation and development of various cancers. Currently, no associations had been revealed between ADAMTS14 and clear cell renal cell carcinoma (ccRCC). Hence, this study was designed to assess the prognostic values and immunological roles of ADAMTS14 in ccRCC and to reveal its potential mechanisms.

**Methods:**

ADAMTS14-related expression profiles and related clinical data were downloaded from The Cancer Genome Atlas (TCGA) dataset, validated by the ICGC dataset, qRT-PCR, and immunohistochemistry. We utilized gene set enrichment analysis (GSEA) to find potentially ADAMTS14-related pathways and applied univariate/multivariate Cox regression analyses to identify independent factors significantly related to overall survival (OS) for ccRCC. A nomogram consisted of independent prognostic factors was also conducted. We further explored the associations between ADAMTS14 with immunity and revealed its potential mechanisms.

**Results:**

ADAMTS14 displayed a higher expression in ccRCC tumor than in adjacent normal tissues, and further validated results of the ICGC dataset; qRT-PCR and immunohistochemistry remained consistent (all *p* < 0.05). Moreover, elevated ADAMTS14 expression was significantly associated with poor OS (*p* < 0.001). Through univariate/multivariate Cox regression analyses, ADAMTS14 was found to be an independent prognostic factor for ccRCC (both *p* < 0.05) and GSEA identified several signaling pathways including INSULIN, MTOR, and PPAR pathways. The nomogram based on independent prognostic factors was successfully established and well evaluated. Moreover, the expression of ADAMTS14 was remarkably associated with immune checkpoint molecules, tumor mutational burden (TMB), immune cells, and tumor immune microenvironment (all *p* < 0.05). Results from TIDE and TCIA showed that highly expressed ADAMTS14 could predict worse efficacy of immunotherapy (all *p* < 0.05). As for its potential mechanisms, we also revealed several LncRNA/RNA binding protein (RBP)/ADAMTS14 mRNA networks.

**Conclusions:**

ADAMTS14 was found to play oncogenic roles in ccRCC and to be significantly associated with immunity. Several LncRNA/RBP/ADAMTS14 mRNA networks were also identified for its potential mechanisms.

## Introduction

Renal cancer is one of the most common malignant tumors in the urinary system, and it is estimated that renal cancer shall have 13,780 newly estimated death and 76,080 newly estimated cases in America in 2021 (1). Based on its histological types, clear cell renal cell carcinoma (ccRCC) remains the most common type and accounts for 70%–80% of all renal cell carcinoma (2). Moreover, the percentages of ccRCC patients diagnosed with localized disease or distant metastasis are often over 30% (3). Although there had been important advances in cancer diagnosis and therapies, 5-year survival rate of metastatic ccRCC patients after surgery still did not exceed 20% (4). As a result, the importance of identifying novel therapeutic targets and effective clinical biomarkers for ccRCC treatment is particularly emphasized, especially in the era of cancer precision medicine (5, 6). Moreover, it is also vital to further investigate the potential mechanisms involved in ccRCC.

A disintegrin-like and metallopeptidase with thrombospondin type 1 motif, also called ADAMTS for short, are enzymes belonging to the extracellular zinc metalloprotease family in humans. Accumulating evidence suggested that ADAMTS proteases were highly associated with arthritis (7, 8), atherosclerosis (9), fertility, and cancer (10–13). As one of the ADAMTS zinc-dependent protease family, ADAMTS14 is located on chromosome 10q22.1 (14–17), having a C-terminal auxiliary domain and an N-terminal catalytic domain to determine the substrate specificity. It had been recently reported that the polymorphism of ADAMTS14 could have multiple effects, including joint effects of ADAMTS14 polymorphisms and environmental mutagens (smoking and betel nut chewing) leading to the tumorigenesis of oral cancer (18) and an involvement of ADAMTS14 polymorphisms in the progression of hepatocellular carcinoma (19). Moreover, the expression of ADAMTS14 had been shown to be markedly upregulated in human breast cancer (20). However, little was known about the relationships between ADAMTS14 and ccRCC. Hence, in this article, we not only assessed the prognostic roles of ADAMTS14 in ccRCC, but also revealed its associations with immunity. As a potential biomarker, we utilized ADAMTS14 to predict the immune responses of immunotherapy. Gene set enrichment analysis (GSEA) and Gene Ontology (GO) analyses were conducted to identify ADAMTS14-related signaling pathways and biological function. In terms of its potential mechanisms, the LncRNA/RNA binding protein (RBP)/ADAMTS14 mRNA networks were also explored for further in-depth analysis. Our outcomes were expected to provide novel treatment targets and effective clinical biomarkers for ccRCC.

## Materials and Methods

### Data Acquisition and Processing

Gene transcriptome profiles and related clinical data of ccRCC patients were downloaded from The Cancer Genome Atlas (TCGA; http://cancergenome.nih.gov/) website, containing 539 ccRCC samples and 72 adjacent normal tissue specimens. Subsequently, we excluded the cases that lack key clinical information and matched the ADAMTS14 gene matrix with corresponding clinical information for further analyses. All analyses were carried out by applying the R software (https://www.r-project.org/). The R package “Limma” was used to evaluate the different expression level of ADAMTS14 mRNA in TCGA ccRCC patients. Ultimately, the cutoff criteria of differently expressed genes were set as the adjusted *p*-value (FDR) <0.05 and |log2 fold change (FC)| ≥1.

### Functional Pathway Enrichment in ccRCC by GSEA and GO Analysis

To seek out potentially ADAMTS14-related pathways, GSEA (21) was applied with the help of the gene set “c2.cp.kegg.v7.1.symbols.gmt” of Kyoto Encyclopedia of Genes and Genomes (KEGG) (22). Moreover, it was considered statistically significant, when the |normalized enrichment score (NES)| >1.5 and the nominal *p*-value < 0.05. To further investigate possible molecular functions of ADAMTS14 in ccRCC, GO analysis was used for further analysis, including molecular function (MF), cellular component (CC), and biological process (BP).

### Univariate/Multivariate Cox Regression Analyses

To further investigate the relationship between ADAMTS14 and overall survival (OS), we performed univariate and multivariate Cox regression analyses to find whether or not ADAMTS14 and other clinical characteristics (staged M; gender; staged T; race; age; grade; staged N; stage) could serve as independent factors related to OS for ccRCC patients in TCGA database, with the threshold of *p*-value < 0.05 (23).

### Validation of the Expression of ADAMTS14 by Immunohistochemical Staining

In order to make our results more credible at the histological level, we collected a total of 10 pairs of tissues from ccRCC patients who underwent radical nephrectomy from the Affiliated Hospital of Nantong University to conduct immunohistochemistry. In addition, we questioned all of these patients and all of them denied having received special treatment like medicine, radiotherapy, and immunotherapy before nephrectomy. Then, we performed routine pathology and immunohistochemistry on the cancer and adjacent normal tissue specimens of ccRCC patients to confirm pathological types. The ADAMTS14 antibody stained in immunohistochemistry was from Abcam (ab198885). After removing the paraffin, hydrating, and blocking, we added the specimen to the anti-ADAMTS14 goat polyclonal antibody (diluted at a ratio of 1:100), and incubated overnight at 4°C. By the application of microscope, we evaluated all sections by comparing the staining between each kidney cancer and adjacent specimens.

### Validation of the Expression of ADAMTS14 by Quantitative Real-Time PCR

In order to obtain total RNA from ccRCC cells, we firstly added 1 ml of Trizol reagent (Life Technology, USA) to the cell culture flask of HK-2, 769-P, and CAKI-1 at ice-cold temperatures, and then applied the Thermo Scientific K1622 Revert Aid First Strand cDNA Synthesis Kit. Finally, qRT-PCR was implemented by ABI QuantStudio5 Real-Time PCR System with the measurement of Fast SYBR^®^ Green Master Mix and calculated by the 2^-ΔΔCt^ method. All relevant primers in this study were acquired from Ribobio (Guangzhou, China), containing ADAMTS14 (F 5’-TCTGAAAGCTGCACACTGCT-3’, R 5’-RGGACCAAGCACCAGAAACAT-3’) and β-actin (F 5’-ATGACTTAGTTGCGTTACACC-3’, R 5’-GACTTCCTGTAACAACGCATC-3’).

### The Establishment of a Prognostic Nomogram

To further forecast the OS of ccRCC patients, we constructed an effective nomogram by utilizing the R package “rms”, and it consists of independent prognostic factors. After dividing points to every factor, we summed up all parameters’ points to calculate total points. Moreover, we applied the concordance index (C-index), receiver operating characteristic (ROC) curves, and calibration curves to evaluate the performance of our established nomogram (24).

### Correlation Analyses of ADAMTS14 and Tumor Neoantigen Burden, Tumor Mutational Burden, and Microsatellite Instability

As previously described (25), we marked tumors as MSI if more than two markers of all five showed the MSI to identify autosomal microsatellite tracts by MISA (http://pgrc.ipk-gatersleben.de/misa/misa.html). Moreover, based on the TCGA database, we calculated the TMB by comparing the gene mutation data between tumor tissues and adjacent normal tissue specimens (26). Moreover, we performed HLA without changing the original settings to detect the TNB based on the TCGA expression dataset (27). All these mentioned analyses were conducted by applying online Sangerbox tools (http://www.sangerbox.com/tool).

### Correlation Analysis of ADAMTS14 and Immune Cell Infiltration and Tumor Microenvironment

Correlation analyses between ADAMTS14 and six different immune cell infiltration levels were carried out by TIMER website (https://cistrome.shinyapps.io/timer/) (28). We implemented the ESTIMATE algorithm to assess the relationships between ADAMTS14 and tumor microenvironment, including the Immune, ESTIMATE, and Stromal scores (29). Furthermore, immune cells, mismatch repair proteins, and immune checkpoint molecules were separately assessed based on the expression levels of ADAMTS14 in TCGA database. Moreover, they were performed using the online Sangerbox tools as well.

### Prediction of ADAMTS14-Related Immune Responses of Immunotherapy

We applied the Tumor Immune Dysfunction and Exclusion (TIDE; http://tide.dfci.harvard.edu/) to evaluate the potential clinical effects of immunotherapy in the groups with different ADAMTS14 expression. Moreover, the higher the TIDE score, the higher the possibility of immune exclusion, indicating a lower probability of patients benefiting from immunotherapy (30). The immunophenoscores of ccRCC patients from The Cancer Immunome Atlas (TCIA; https://tcia.at/) database were also utilized to assess the immune properties of ADAMTS14 in ccRCC (31).

### Identification of Potential Mechanisms of LncRNA/RBP/ADAMTS14 mRNA Networks

To further explore the potential mechanisms of ADAMTS14 in ccRCC, we revealed several LncRNA/RBP/ADAMTS14 mRNA networks. Firstly, starBase v2.0 was utilized to predict ADAMTS14 targeted RBPs, with the threshold of strict stringency (≥5) and pan-Cancer ≥10 cancer types. After that, we used starBase database v2.0 once again to predict a selected RBP-targeted LncRNAs, with the threshold of strict stringency (≥5), pan-Cancer ≥10 cancer types, hub LncRNAs in TCGA ccRCC (*p*-value < 0.05, |log2 FC| ≥ 1 and FDR < 0.05), and LncRNAs positively correlated with ADAMTS14 in TCGA ccRCC (corFilter = 0.3 and pFilter = 0.001). Finally, the LncRNA/RBP/ADAMTS14 mRNA networks were identified and visualized by Cytoscape 3.6.1 software.

## Results

### ADAMTS14 mRNA Expression in ccRCC and Validation by ICGC Dataset and qRT-PCR

**Figure 1:**
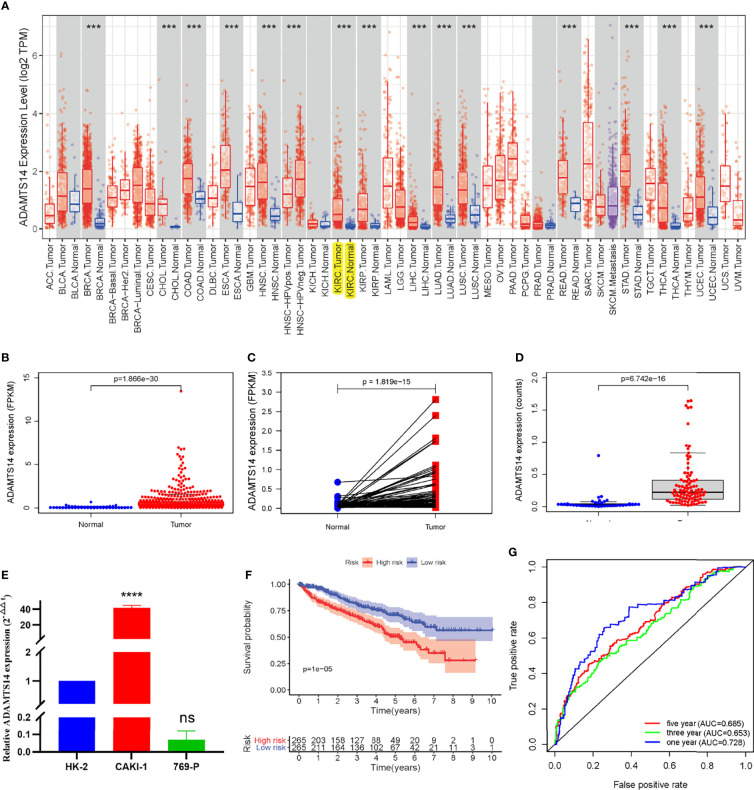
The ADAMTS14 mRNA expression levels in ccRCC. **(A)** The mRNA expression levels of ADAMTS14 in pan-Cancer from TCGA datasets. **(B)** Boxplot of the ADAMTS14 mRNA expression (FPKM) in TCGA ccRCC dataset (*T* = 539, *N* = 72). **(C)** Pairwise boxplot of the ADAMTS14 mRNA expression (FPKM) in TCGA ccRCC dataset (*T* = 72, *N* = 72). **(D)** Boxplot of the ADAMTS14 mRNA expression (counts) in the ICGC dataset (*T* = 91, *N* = 45). **(E)** qRT-PCR results of ADAMTS14 detected from ccRCC cells (2^-ΔΔCt^ method). **(F)** Kaplan–Meier survival curve of ADAMTS14 in TCGA ccRCC dataset. **(G)** ROC curves associated with 1-, 3-, and 5-year AUC values of ADAMTS14 in TCGA ccRCC dataset. ***P < 0.001; ****P < 0.0001; ns, not significant.

ADAMTS14 mRNA expression levels in pan-Cancer from TCGA are displayed in **Figure 1A**. Moreover, detailed ccRCC sample information from TCGA was shown in **Supplementary Table 1** and the mRNA expression levels of ADAMTS14 in pan-Cancer from TCGA and GTEx datasets are shown in **Supplementary Figure 1**, which contains more adjacent normal tissue specimens. As shown in **Figure 1B**, ADAMTS14 displayed a higher expression in ccRCC tumor tissues than in adjacent normal tissues (*p*-value <0.001). Pairwise boxplot and ICGC dataset boxplot suggested similar results (both *p*-values <0.001, **Figures 1C, D**). The outcomes of qRT-PCR presented that the relative ADAMTS14 mRNA levels were highly expressed in CAKI-1 (renal cancer cell lines; *p*-value <0.0001), while it was not significantly downregulated in 769-P (renal cancer cell lines; *p*-value *>* 0.05), compared with HK-2 (normal kidney cell lines) by the 2^-ΔΔCt^ method (**Figure 1E**). In addition, we examined the expression patterns of ADAMTS14 in various cancer cell lines in the CCLE database containing renal cancer (**Supplementary Figure 2**). According to the median expression, ccRCC patients were classified into high- and low-risk subclasses and the KM survival curves shed light on the fact that the low-ADAMTS14 groups could have a better OS than the high-ADAMTS14 groups (*p*-value <0.001; **Figure 1F**). To further evaluate the diagnostic power of ADAMTS14, we conducted a ROC analysis and the 1-year, 3-year, and 5-year area under the curve (AUC) values were 0.728, 0.653, and 0.685, respectively, indicating a moderate diagnostic effect in diagnosing ccRCC based on the expression of ADAMTS14 (**Figure 1G**). All in all, ADAMTS14 was highly expressed in ccRCC and correlated with poor prognosis, suggesting that this gene may be a key gene in ccRCC.

### Verification of the ADAMTS14 Protein Expression by Immunohistochemistry

**Figure 2:**
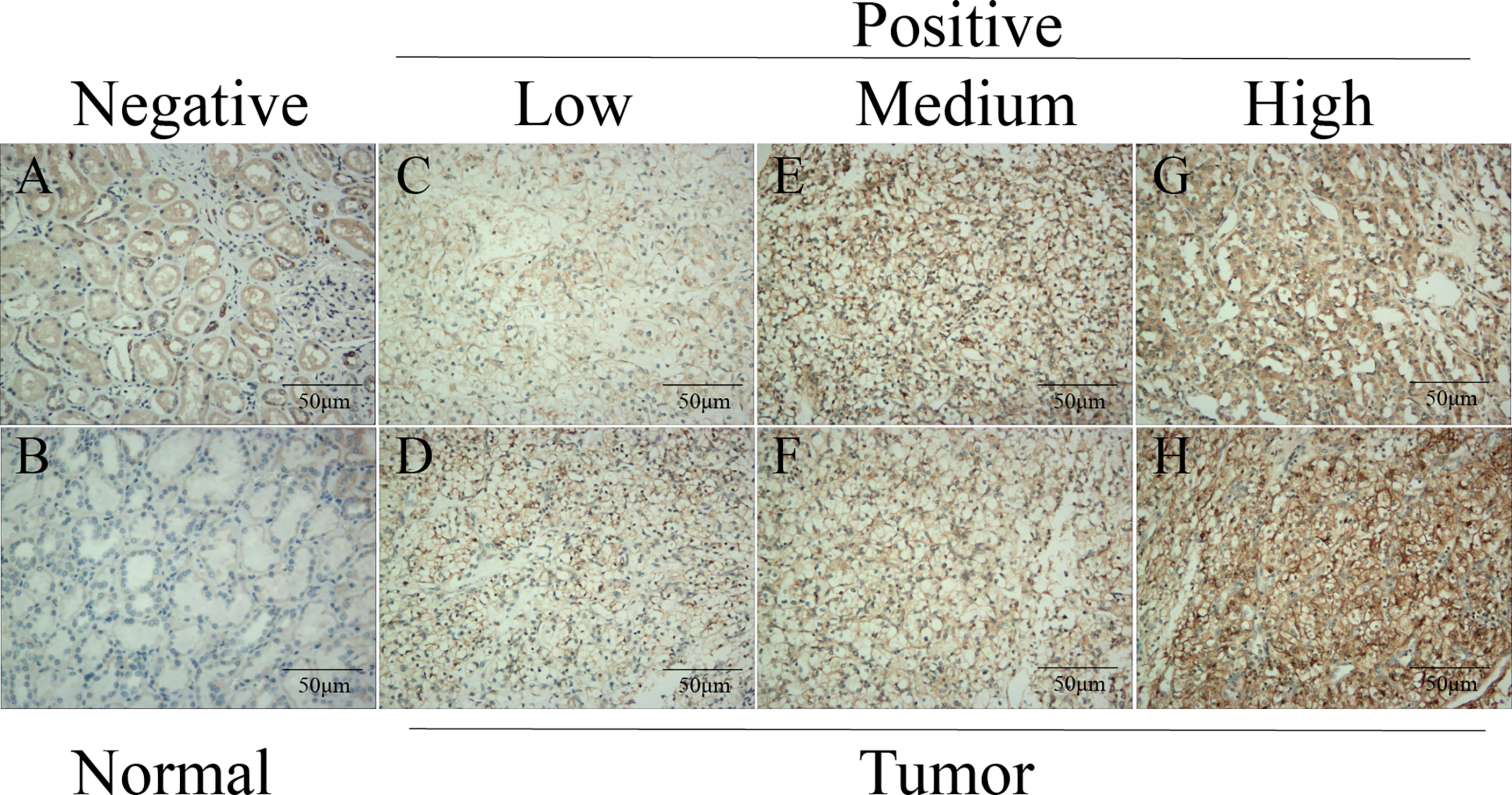
Validation of the protein expression levels of ADAMTS14 by immunohistochemistry in ccRCC tissue specimens. **(A, B)** Negative expression of ADAMTS14 in adjacent normal tissue specimens of ccRCC patients. **(C, D)** Low expression of ADAMTS14 in ccRCC tissues. **(E, F)** Medium expression of ADAMTS14 in ccRCC tissues. **(G, H)** High expression of ADAMTS14 in ccRCC tissues; scale bar = 50 μm.

To further verify the ADAMTS14 protein expression level of tissues in ccRCC, we selected a total of 10 patients undergoing radical nephrectomy from the Affiliated Hospital of Nantong University in the past 2 years. Then, we performed routine pathology and immunohistochemistry on the cancer and para-cancerous tissue samples of ccRCC patients. As detailed in **Figure 2**, ADAMTS14 protein was positively expressed in ccRCC cancer tissues than in adjacent normal tissue specimens, and the numbers of patients included in each of the low-, medium-, and high-ADAMTS14 groups were 3, 3, and 4, respectively. Consistent with the mRNA expression levels of ADAMTS14, its protein expression levels were also elevated in ccRCC tumor tissues.

### Distributions of ADAMTS14 Expression in Clinicopathologic Variables

**Figure 3:**
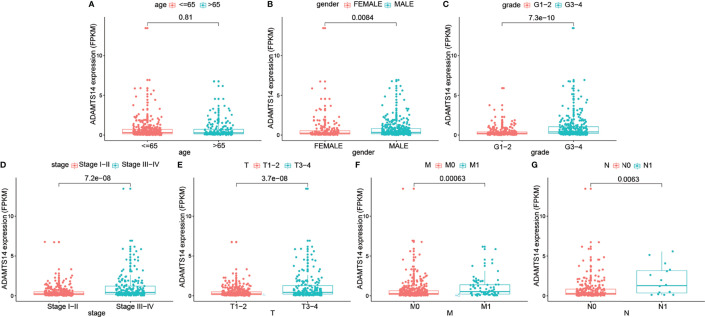
Associations between the ADAMTS14 expression (FPKM) and (**A**) age, **(B)** gender, **(C)** grade, **(D)** stage, **(E)** staged T, **(F)** staged M, and **(G)** staged N in TCGA ccRCC dataset.

As shown in **Figure 3**, elevated ADAMTS14 expression was significantly linked to gender (*p*-value = 0.0084), grade (*p*-value < 0.001), stage (*p*-value < 0.001), staged T (*p*-value < 0.001), staged M (*p*-value < 0.001), and staged N (p-value = 0.0063), while it was not related to age (*p*-value = 0.81). All of the abovementioned results suggested that ccRCC patients with elevated ADAMTS14 expression might be more likely to progress to advanced stages and develop metastasis, compared with patients with low ADAMTS14 expression.

### ADAMTS14 Might Be an Independent Prognostic Factor for ccRCC

We performed univariate and multivariate Cox regression analyses to identify independent factors highly related to the OS for ccRCC from ADAMTS14 and other clinical characteristics (staged M, gender, staged T, race, age, grade, staged N, and stage). Our outcomes showed that stage (HR = 1.914, *p*-value < 0.001), grade (HR = 1.338, *p*-value = 0.013), age (HR = 1.038, *p*-value < 0.001), and ADAMTS14 (HR = 1.185, *p*-value < 0.001) were finally screened out (**Supplementary Figure 3** and **Table 1**). Hence, four independent prognostic factors related to ccRCC were identified, consisting of stage, grade, age, and ADAMTS14.

**Table 1:** Associations between ADAMTS14, clinicopathologic characteristics, and OS in TCGA ccRCC patients by univariate and multivariate cox analysis.

Clinical characteristics	Univariate analysis		Multivariate analysis	
	HR (95% CI)	*P*-value	HR (95% CI)	*P-*value
Age	1.033 (1.020 - 1.047)	**<0.001**	1.038 (1.023 - 1.053)	**<0.001**
Gender	0.933 (0.680 - 1.282)	0.670	0.995 (0.716 - 1.384)	0.978
Race	1.193 (0.716 - 1.988)	0.498	1.065 (0.618 - 1.833)	0.821
Grade	1.967 (1.639 - 2.361)	**<0.001**	1.338 (1.064 - 1.681)	**0.013**
Stage	1.856 (1.644 - 2.095)	**<0.001**	1.914 (1.360 - 2.694)	**<0.001**
T	1.998 (1.689 - 2.362)	**<0.001**	1.004 (0.757 - 1.332)	0.979
M	2.100 (1.661 - 2.655)	**<0.001**	0.735 (0.398 - 1.358)	0.326
N	0.863 (0.739 - 1.008)	0.063	0.894 (0.759 - 1.052)	0.177
ADAMTS14	1.256 (1.175 - 1.343)	**<0.001**	1.185 (1.085 - 1.294)	**<0.001**

Bold values indicated P<0.05.

### Nomogram Establishment for ccRCC

**Figure 4:**
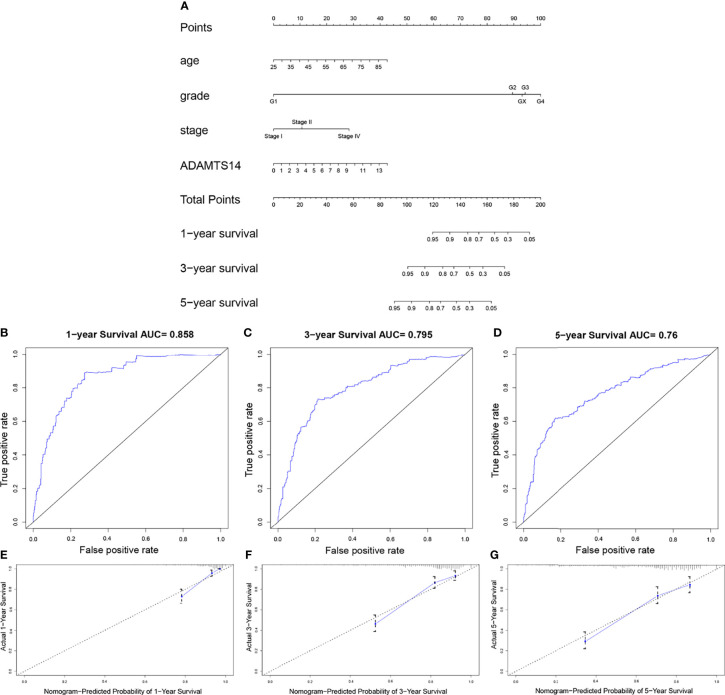
Establishment of a prognostic prediction nomogram for ccRCC in TCGA dataset. **(A)** Nomogram for predicting 1-, 3-, and 5-year OS for ccRCC patients in TCGA. **(B–D)** One-, 3-, and 5-year ROC curves of the established nomogram. **(E–G)** One-, 3-, and 5-year calibration plots of the established nomogram.

To forecast the OS prognosis of ccRCC patients, we constructed an effective four-factor nomogram, based on the results of multivariate Cox analysis (**Figure 4A**). C-index and ROC curves associated with AUC values of 1, 3, and 5 years were 0.780, 0.858, 0.795, and 0.76, respectively, showing a moderate performance (**Figures 4B–D** and **Table 2**). Calibration plots of 1, 3, and 5 years also suggested a consistency of actual and predicted results (**Figures 4E–G**). In summary, we successfully constructed a well-performing nomogram plot and predicted the prognosis of ccRCC patients well.

**Table 2:** The 1-, 3-, 5-year AUCs and C-index of our constructed nomogram.

	1-year	3-year	5-year	C-index
AUC	0.858	0.795	0.760	0.780

### Signaling Pathways and Gene Function Related to ADAMTS14

**Figure 5:**
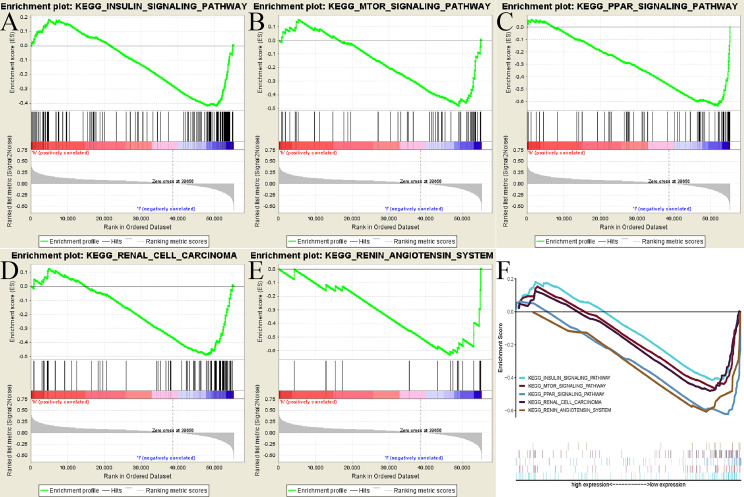
GSEA identified ADAMTS14-related signaling pathways in TCGA dataset. **(A)** INSULIN signaling pathway. **(B)** MTOR signaling pathway. **(C)** PPAR signaling pathway. **(D)** Renal cell carcinoma signaling pathway. **(E)** Renin–angiotensin system signaling pathway. **(F)** All of these five significantly enriched signaling pathways.

To seek out potentially ADAMTS14-related pathways, GSEA [21] was applied with the help of the gene set “c2.cp.kegg.v7.1.symbols.gmt”. According to the threshold of the |NES| >1.5 and nominal *p*-value < 0.05, we identified five signaling pathways that showed significantly different enrichment in the ADAMTS14 expression phenotype, including INSULIN, MTOR, PPAR, renal cell carcinoma, and renin–angiotensin system pathways (**Figure 5** and **Table 3**). We further performed Gene Ontology (GO) analysis including molecular function (MF), cellular component (CC), and biological process (BP) in **Supplementary Figure 4**. As shown in **Supplementary Figure 4**, ADAMTS14 was also proved to be significantly associated with immunity. Overall, biological functions and possibly signaling pathways associated with ADAMTS14 are revealed by our results, which were instructive for subsequent functional studies of ADAMTS14 in ccRCC.

**Table 3:** Results of gene set enrichment analysis.

MSigDB collection	Gene set name	NES	NOM p-val	FDR q-val
c2.cp.kegg.v7.1symbols.gmt	INSULIN_SIGNALING_PATHWAY	-1.773	0.015	0.048
	MTOR_SIGNALING_PATHWAY	-1.768	0.010	0.048
	PPAR_SIGNALING_PATHWAY	-2.077	0.004	0.014
	RENAL_CELL_CARCINOMA	-1.675	0.031	0.072
	RENIN_ANGIOTENSIN_SYSTEM	-1.866	0.008	0.035

### Associations Between ADAMTS14 and Protein–Protein Interaction, TNB, TMB, and MSI

**Figure 6:**
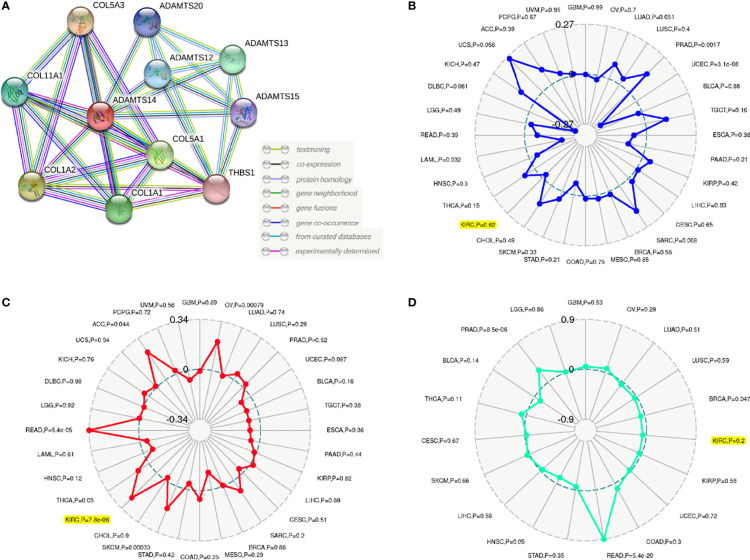
Associations between ADAMTS14 and **(A)** PPI, **(B)** MSI, **(C)** TMB, and **(D)** TNB in TCGA dataset.

Results of the PPI network suggested that a total of ten genes (COL5A3, COL11A1, COL1A2, COL5A1, COL1A1, THBS1, ADAMTS12, ADAMTS13, ADAMTS15, and ADAMTS20) were significantly related to ADAMTS14 (**Figure 6A**). Our outcomes also shed light on the fact that ADAMTS14 was significantly linked to TMB (*p*-value <0.001), while it was not related to MSI (*p*-value = 0.62) or TNB (*p*-value = 0.2) (**Figures 6B–D**). All in all, TMB might be a vital factor involved in the roles of ADAMTS14 in ccRCC.

### Relationships Among ADAMTS14 and Tumor Microenvironment, Methyltransferase, and Immune Infiltrations

**Figure 7:**
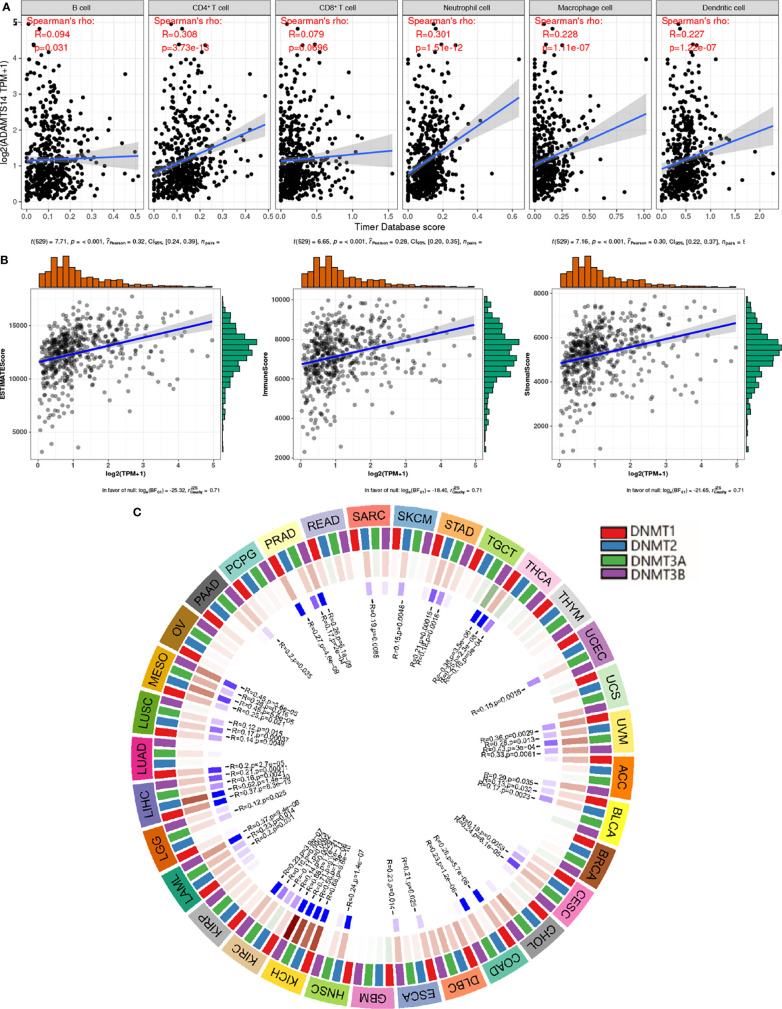
Associations between ADAMTS14 and **(A)** immune infiltrations, **(B)** tumor microenvironment, and **(C)** methyltransferase in TCGA dataset.

Based on the cutoff values of correlation coefficient greater than 0.3 and *p*-value less than or equal to 0.001, we investigated the potential relationships between ADAMTS14 and six immune cells’ infiltration levels and found that CD4+ T cells and neutrophil cells were significantly correlated with ADAMTS14 expression, while other immune cells were not (**Figure 7A**). As for the tumor microenvironment, significant results were also shown between ADAMTS14 and ESTIMATE, Stromal, and Immune scores (**Figure 7B**). Moreover, the ADAMTS14 expression was highly correlated to methyltransferases including DNA methyltransferase 2 (DNMT2), DNA methyltransferase 3A (DNMT3A), DNA methyltransferase 1 (DNMT1), and DNA methyltransferase 3B (DNMT3B) in the diverse tumors especially in ccRCC (all *p*-values < 0.01, **Figure 7C**). Thus, ADAMTS14 was highly related to immunity and methyltransferases, providing potential guidance for immunotherapy.

### Associations Between ADAMTS14 and Immune Checkpoint Molecules, Mismatch Repair Proteins, and Immune Cells

**Figure 8:**
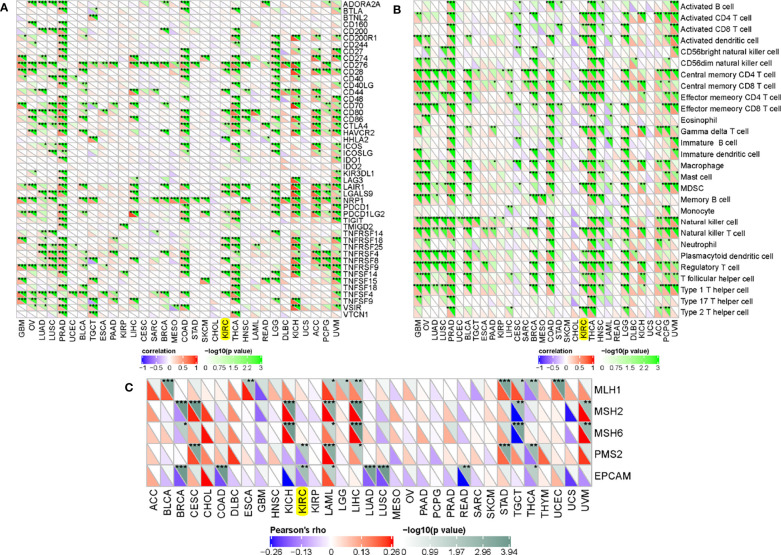
Associations between ADAMTS14 and **(A)** immune checkpoint molecules, **(B)** immune cells, and **(C)** mismatch repair proteins in TCGA dataset. *p < 0.05; **p < 0.01; ***p < 0.001.

To further analyze the correlations between ADAMTS14 and immune checkpoint molecules, our results showed that ADAMTS14 were markedly related to immune checkpoint molecules in ccRCC such as CD28, CD44, CD276, CD80, and CTLA4 (all *p*-values < 0.05, **Figure 8A**). Moreover, ADAMTS14 was highly associated with several immune cells in ccRCC, such as activated dendritic cell, central memory CD8 T cell, central memory CD4 T cell, and activated CD4 T cell (all *p*-values < 0.05, **Figure 8B**). As for mismatch repair proteins, ADAMTS14 was potentially associated with PMS2 and EPCAM mismatch repair proteins in ccRCC (both *p*-values < 0.01, **Figure 8C**). All in all, ADAMTS14 was discovered to be remarkably related to immune checkpoint molecules, mismatch repair proteins, and immune cells.

### Prediction of ADAMTS14-Related Immune Responses of Immunotherapy

**Figure 9:**
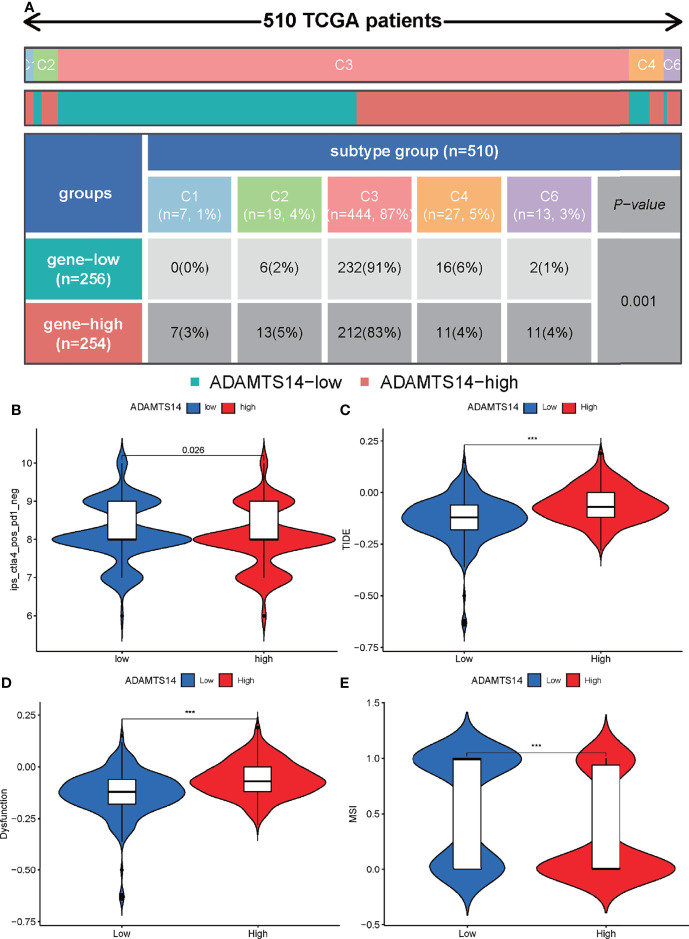
Prediction of ADAMTS14-related immune responses of immunotherapy in ccRCC patients. **(A)** Distribution of ADAMTS14 expression in pan-cancer immune subtypes in TCGA dataset. **(B)** Distribution of ADAMTS14 expression in CTLA4 scores by TCIA dataset. **(C)** Distribution of ADAMTS14 expression in TIDE scores by TIDE dataset. **(D)** Distribution of ADAMTS14 expression in T-cell dysfunction scores by TIDE dataset. **(E)** Distribution of ADAMTS14 expression in MSI scores by TIDE dataset. ***p < 0.001.

As for pan-cancer immune subtypes, our results indicated that ADAMTS14 was differently expressed in pan-cancer immune subtypes of C1, C2, C3, C4, and C6 (*p*-value = 0.001, **Figure 9A**). The immunophenoscores of ccRCC patients from TCIA database (https://tcia.at/) were also utilized to assess the immune properties of ADAMTS14 in ccRCC (**Figure 9B** and **Supplementary Figure 5**). Our results indicated that the low- and high-ADAMTS14 groups had significant immunogenicity for CTLA4 immunotherapy (*p*-value = 0.026), but not for PD1 immunotherapy (*p*-value = 0.075). Moreover, we applied the TIDE database to evaluate the potential clinical effects of immunotherapy in groups with different ADAMTS14 expression. As shown in **Figures 9C–E**, high expression of ADAMTS14 had a higher TIDE score, a higher T-cell dysfunction score, and a lower MSI score, indicating that these patients might have a lower efficacy and worse outcome after accepting the immunotherapy than ccRCC patients with low expression of ADAMTS14. In conclusion, our results demonstrated the predicted response of ccRCC patients when receiving immunotherapy. On the one hand, ccRCC patients with high ADAMTS14 expression shall have a poor prognosis when receiving immunotherapy. On the other hand, CTLA4 might be an effective target for ccRCC patients with low ADAMTS14 expression.

### Identification of LncRNA/RBP/ADAMTS14 mRNA Networks

**Figure 10:**
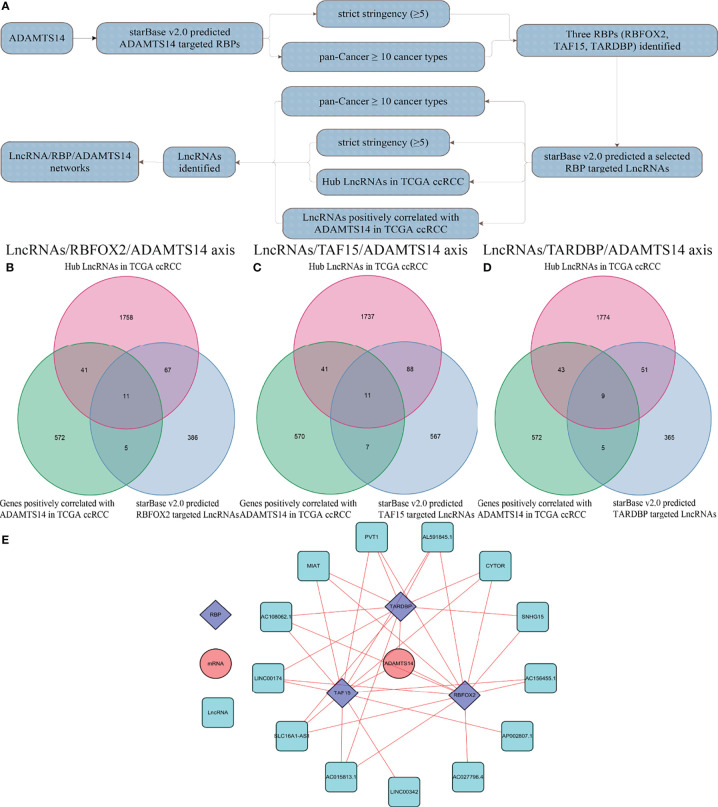
Identification of LncRNA/RBP/ADAMTS14 mRNA networks in ccRCC patients from TCGA dataset. **(A)** The whole flow chart of identification. **(B)** Venn diagrams of LncRNAs/RBFOX2/ADAMTS14 axes. **(C)** Venn diagrams of LncRNAs/TAF15/ADAMTS14 axes. **(D)** Venn diagrams of LncRNAs/TARDBP/ADAMTS14 axes. **(E)** LncRNA/RBP/ADAMTS14 mRNA networks by Cytoscape 3.6.1.

To reveal potentially ADAMTS14-related mechanisms in ccRCC, we presented several LncRNA/RBP/ADAMTS14 mRNA networks. As detailed in **Figure 10A**, starBase v2.0 was firstly utilized to predict ADAMTS14-targeted RBPs, with the threshold of strict stringency (≥5) and pan-Cancer ≥ 10 cancer types. After identifying the potential RBPs, starBase v2.0 was utilized once again to predict selected RBP-targeted LncRNAs, with the threshold of strict stringency (≥5), pan-Cancer ≥ 10 cancer types, hub LncRNAs in TCGA ccRCC (*p*-value < 0.05, |log2 FC| ≥ 1, and FDR < 0.05), and LncRNAs positively correlated with ADAMTS14 in TCGA ccRCC (corFilter = 0.3 and pFilter = 0.001). **Figures 10B–D** were utilized to identify selected RBP-targeted LncRNAs by Venn diagrams. Three Venn diagrams represent three RBP-involved axes (the LncRNAs/RBFOX2/ADAMTS14 axis, the LncRNAs/TAF15/ADAMTS14 axis, and the LncRNAs/TARDBP/ADAMTS14 axis) to find RBP-targeted LncRNAs. Finally, the potential LncRNA/RBP/ADAMTS14 mRNA networks were identified and visualized by Cytoscape 3.6.1 software in **Figure 10E**.

## Discussion

The number of deaths caused by renal cancer was more than 10,000 each year in the United States (32). Despite the early detection and surgical treatment, postoperative recurrence is still common and RCC patients’ prognosis remains poor. ADAMTS14, as one member of the ADAMTS zinc-dependent protease family, had been found to be associated with the high number of diseases. Recently, it had been found to play vital roles in the regulation of collagen and formation of lymphatic vessels (33, 34). Moreover, it was also revealed to be significantly associated with oral, hepatocellular, and colon cancers (35–37). However, little was known about the relationships between ADAMTS14 and ccRCC. Hence, the present study was designed to evaluate the prognostic and immunological values of ADAMTS14 in ccRCC and explore its potential mechanisms of LncRNA/RNA binding protein (RBP)/ADAMTS14 mRNA networks.

In this study, our results indicated that ADAMTS14 displayed a higher expression in ccRCC tumor tissues than in adjacent normal tissue specimens, associated with poor OS. Its expressions were also verified by the ICGC dataset, qRT-PCR, and immunohistochemistry. ADAMTS14 mRNA expression was also revealed to be dramatically correlated with gender, staged N, grade, staged M, stage, and staged T. By means of univariate/multivariate Cox regression analyses, our results discovered that ADAMTS14 could be an independent prognostic factor for ccRCC. GSEA was utilized to find ADAMTS14-related signaling pathways including INSULIN, MTOR, and PPAR pathways. A nomogram consisting of independent prognostic factors including ADAMTS14 was also conducted to predict the ccRCC patients’ 1-, 3-, and 5-year survival prognosis. Moreover, we shed light on the fact that the expression of ADAMTS14 was remarkably related to immune cells, TMB, immune checkpoint molecules, and tumor immune microenvironment. By means of TIDE and TCIA, we found that highly expressed ADAMTS14 could predict worse efficacy of immunotherapy. As for its potential mechanisms, we also revealed several LncRNA/RBP/ADAMTS14 mRNA networks.

As reported by previously published articles, the ADAMTS14 gene was discovered to play critical roles in the progress of inflammation. Dupont et al. found that ADAMTS played significant roles in regulating the immune system, through a cross-talk of the TGF-β pathways and mesenchymal cells, in the ADAMTS2–ADAMTS14-deficient mice (38). Ma et al. found the associations between ADAMTS14 gene polymorphism and knee osteoarthritis (KOA), offering a therapeutic target and diagnostic marker for treatment of KOA (39). Wang et al. shed light on the significant correlations between ADAMTS14 rs4747096 polymorphism and the osteoarthritis of the temporomandibular joint in Chinese Han women (40). As for the risk in the aspect of cancer, Alonso et al. presented that disrupting the ADAMTS14 methylation patterns might lead to predictive biomarkers for colorectal cancer (37). Sheu et al. suggested an involvement of ADAMTS14 polymorphisms in the progression of hepatocellular carcinoma (19). Moreover, the expression of ADAMTS14 had been shown to be markedly upregulated in human breast cancer (20). Basically, consistent with previous articles, our results also indicated that ADAMTS14 had a higher expression in ccRCC tissues than in adjacent normal tissue specimens and elevated ADAMTS14 expression was also significantly associated with poor OS.

As reported by Lin et al, decreased cytoplasmic ADAMTS14 expression levels were found to be involved in oral squamous cell carcinoma progression and prognosis (36). It was inconsistent with our trends in this article and this situation was well worthy of a discussion and analysis. After carefully reviewing their experiment, we found that the opposite results might be due to the following reasons: Firstly, the expression of ADAMTS14 might be very different in variable organs. Secondly, their study had 60.8% and 24% of patients receiving radiotherapy and chemotherapy, being more likely to result in altered gene expression. Overall, there were some differences in experimental orientation and methodology between these two studies and we shall refer to their study to refine our subsequent study of ADAMTS14.

By means of GSEA, we identified five ADAMTS14-related signaling pathways, including MTOR, PPAR, INSULIN signaling pathway, renal cell carcinoma, and renin–angiotensin system. As reported, MTOR signaling pathway was frequently activated to regulate cancer cell growth and metabolism (41). The upregulated PPAR expression could be against nutritional deprivation, thus leading to the improvement of cancer survival and tumor progression (42). Insulin and insulin-like growth factors were reported to play important roles in the progression and development of tumor, including renal cancer (43). Overall, biological functions and possibly signaling pathways associated with ADAMTS14 are revealed by our results, which were instructive for subsequent functional studies of ADAMTS14 in ccRCC.

To intuitively reveal the prognosis of cancer patients, nomograms were often applied in various cancers (44–46). In this study, we constructed an effective nomogram to forecast the OS of ccRCC patients with data from TCGA, based on four independent prognostic factors including stage, grade, age, and ADAMTS14. Then, C-index, ROC curves, and calibration plots exhibited a moderate performance of our established nomogram in predicting OS for ccRCC. In summary, we successfully constructed a well-performing nomogram plot and predicted the prognosis of ccRCC patients well.

For the associations between ADAMTS14 and immunity, our results found that the ADAMTS14 expression was markedly linked to tumor immune microenvironment, immune cells, and immune checkpoint molecules. Therein, immune infiltration and tumor immune microenvironment took important parts in multiple tumors and they were able to predict immune responses of immunotherapy (47–50). Moreover, immune cells as well as immune checkpoint molecules had been regarded as promising targets or pathways for the treatment of various cancers (51–53). Moreover, we applied the TIDE database to evaluate the potential clinical effects of immunotherapy in the groups with different ADAMTS14 expression. Our results found that elevated ADAMTS14 expression had a higher TIDE score, a higher T-cell dysfunction score, and a lower MSI score, indicating that these patients might have a lower efficacy and worse outcome after accepting the immunotherapy than those ccRCC patients with low expression of ADAMTS14. The immunophenoscores of ccRCC patients from TCIA database (https://tcia.at/) were also utilized to assess the immune properties of ADAMTS14 in ccRCC and the low- and high-ADAMTS14 groups had different immunogenicity for CTLA4 immunotherapy. Thus, these findings had very high values for clinical application.

RBPs were reported to be involved in a wide range of mechanisms, mainly containing alternative splicing, polyadenylation, mRNA stability, mRNA localization, and translation (54). Zou et al. found that LINC00324 could promote the cell proliferation of gastric cancer, through binding with RBP (HuR) and stabilizing the mRNA expression of FAM83B (55). Wang et al. reported that lncRNA EGFR-AS1 was able to promote cell growth and metastasis of renal cancer, by means of affecting RBP (HuR)-mediated EGFR mRNA stability (56). Yamada et al. revealed that the miR-29 family regulated genes containing ADAMTS14 were significantly related to renal cancer’s molecular pathogenesis (57). In this article, we also utilized RBP’s function of mRNA stability to identify the LncRNA/RBP/ADAMTS14 mRNA networks for its potential mechanisms, serving as a novel therapeutic strategy of ADAMTS14 for ccRCC.

There were some shining points in this study. On the one hand, the expression of ADAMTS14 was not only discovered in TCGA dataset, but also verified by the ICGC dataset, qRT-PCR, and immunohistochemistry, making our results more credible. On the other hand, we found that ADAMTS14 was significantly associated with immunity and could predict immune responses of immunotherapy by means of TIDE and TCIA. Last but not least, we revealed the LncRNA/RBP/ADAMTS14 mRNA networks for its potential mechanisms. However, there were several limitations too. Firstly, this study lacks treatment information. In addition to tumor biology, there are various factors involved with the ccRCC patients’ survival prognosis, especially the clinical medical data corresponding to a treatment institution. Secondly, we found that elevated ADAMTS14 expression was significantly associated with poor OS. However, GSEA identified several cancer-related signaling pathways including INSULIN, MTOR, and PPAR pathways that were enriched in the low-ADAMTS14 expression group. In fact, these results only suggested that ADAMTS14 was related to these pathways, but their actual associations should be further confirmed by experiments. Thirdly, the role of ADAMTS14 in ccRCC had not been fully explored by experiments. The expression of ADAMTS14 mRNA in tissues and the study of co-expression with immune-related genes, especially the LncRNA/RBP/ADAMTS14 mRNA networks, should be verified in further studies. Lastly, when predicting the mechanisms of LncRNA/miRNA/ADAMTS14 mRNA networks, the information of ADAMTS14 mRNA was absent in the step of miRNA–mRNA targets. We shall further investigate the LncRNA/miRNA/ADAMTS14 mRNA networks when the starBase database is updated in our subsequent articles.

## Conclusions

Taken together, the present study revealed the oncogenic roles of ADAMTS14 in ccRCC. Univariate/multivariate Cox regression analyses were performed to show that ADAMTS14 could be an independent factor for ccRCC and GSEA was applied to find ADAMTS14-associated pathways, including INSULIN, MTOR, and PPAR signaling pathways. We also found that ADAMTS14 was dramatically linked with immunity and could predict immune responses of immunotherapy. In addition, we also identified LncRNA/RBP/ADAMTS14 mRNA networks for its potential mechanisms. Further *in vivo* and *in vitro* experiments were required to validate our results.

## Data Availability Statement

The datasets presented in this study can be found in online repositories. The names of the repository/repositories and accession number(s) can be found in the article/**Supplementary Material**.

## Ethics Statement

The studies involving human participants were reviewed and approved by Institutional Research Ethics Committees of Affiliated Hospital of Nantong University. The patients/participants provided their written informed consent to participate in this study.

## Author Contributions

YC: Manuscript writing/editing/revision. BZ: Manuscript writing/editing. HJ: Manuscript revision. YC: Data collection or management. SL: Data analysis. QX, BZ, and YW: Protocol/project development. All authors contributed to the article and approved the submitted version.

## Funding

This article was funded by the Postdoctoral Science Foundation of Jiangsu Province: 2020Z071 and Nantong Science and Technology Planning Project: JC2021183.

## Conflict of Interest

The authors declare that the research was conducted in the absence of any commercial or financial relationships that could be construed as a potential conflict of interest.

## Publisher’s Note

All claims expressed in this article are solely those of the authors and do not necessarily represent those of their affiliated organizations, or those of the publisher, the editors and the reviewers. Any product that may be evaluated in this article, or claim that may be made by its manufacturer, is not guaranteed or endorsed by the publisher.

## Supplementary Material

Supplementary Figure 1 The mRNA expression levels of ADAMTS14 in pan-Cancer from TCGA and GTEx datasets. ****P* < 0.001;

Supplementary Figure 2 The expression patterns of ADAMTS14 in cell lines from CCLE database; **(A)** Various cancer cell lines; **(B)** Renal cancer cell lines;

Supplementary Figure 3 ADAMTS14 could serve as an independent prognostic factor for ccRCC in TCGA dataset; **(A)** Univariate cox regression analysis; **(B)** Multivariate cox regression analysis;

Supplementary Figure 4 The Gene Ontology (GO) analysis of ADAMTS14 in ccRCC patients in TCGA dataset.

Supplementary Figure 5 Prediction of ADAMTS14 related immune responses of immunotherapy in ccRCC patients by TCIA dataset; **(A)** Distribution of ADAMTS14 expression in scores of CTLA4 negative and PD1 negative; **(B)** Distribution of ADAMTS14 expression in scores of CTLA4 negative and PD1 positive; **(C)** Distribution of ADAMTS14 expression in scores of CTLA4 positive and PD1 positive.

## Abbreviations

ccRCC, Clear cell renal cell carcinoma; RBP, RNA binding protein; TCGA, The Cancer Genome Atlas; GSEA, Gene set enrichment analysis; OS, Overall survival; GO, Gene Ontology; TNB, Tumor neoantigen burden; TMB, Tumor mutational burden; MSI, Microsatellite instability; CHOL, Cholangiocarcinoma; OV, Ovarian serous cystadenocarcinoma; LIHC, Liver hepatocellular carcinoma; ESCA, Esophageal carcinoma; PAAD, Pancreatic adenocarcinoma; STAD, Stomach adenocarcinoma; COAD, Colon adenocarcinoma; KIRC, Kidney renal clear cell carcinoma; READ, Rectum adenocarcinoma; PCPG, Pheochromocytoma and paraganglioma; HNSC, Head and neck squamous cell carcinoma; CESC, Cervical squamous cell carcinoma and endocervical adenocarcinoma; LUSC, Lung squamous cell carcinoma; KIRP, Kidney renal papillary cell carcinoma; KICH, Kidney chromophobe; BRCA, Breast invasive carcinoma; THCA, Thyroid carcinoma; DLBC, Lymphoid neoplasm diffuse large B-cell lymphoma; SKCM, Skin cutaneous melanoma; BLCA, Bladder urothelial carcinoma; SARC, Sarcoma; THYM, Thymoma; LUAD, Lung adenocarcinoma; UCEC, Uterine corpus endometrial carcinoma; UCS, Uterine carcinosarcoma; ACC, Adrenocortical carcinoma; PRAD, Prostate adenocarcinoma.
