# Supplementary figures and images for: Survival Prognosis, Tumor Immune Landscape, and Immune Responses of ADAMTS14 in Clear Cell Renal Cell Carcinoma and Its Potential Mechanisms
Source: Front Immunol. 2022 Apr 29;13:790608. doi: 10.3389/fimmu.2022.790608 (PMC9099013; doi:10.3389/fimmu.2022.790608)

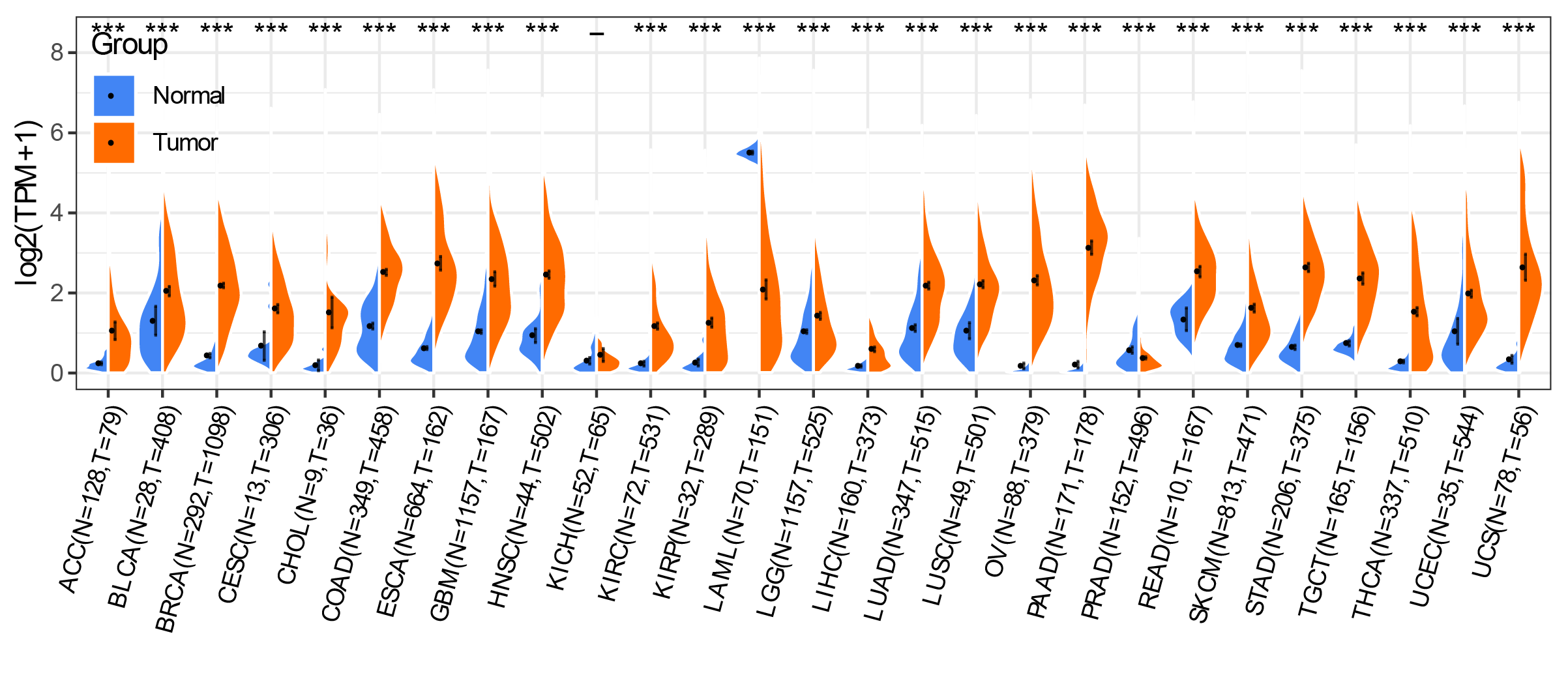

Supplement: Supplementary Figure 1 — The mRNA expression levels of ADAMTS14 in pan-Cancer from TCGA and GTEx datasets. ***P < 0.001; [file Image_1.tif]

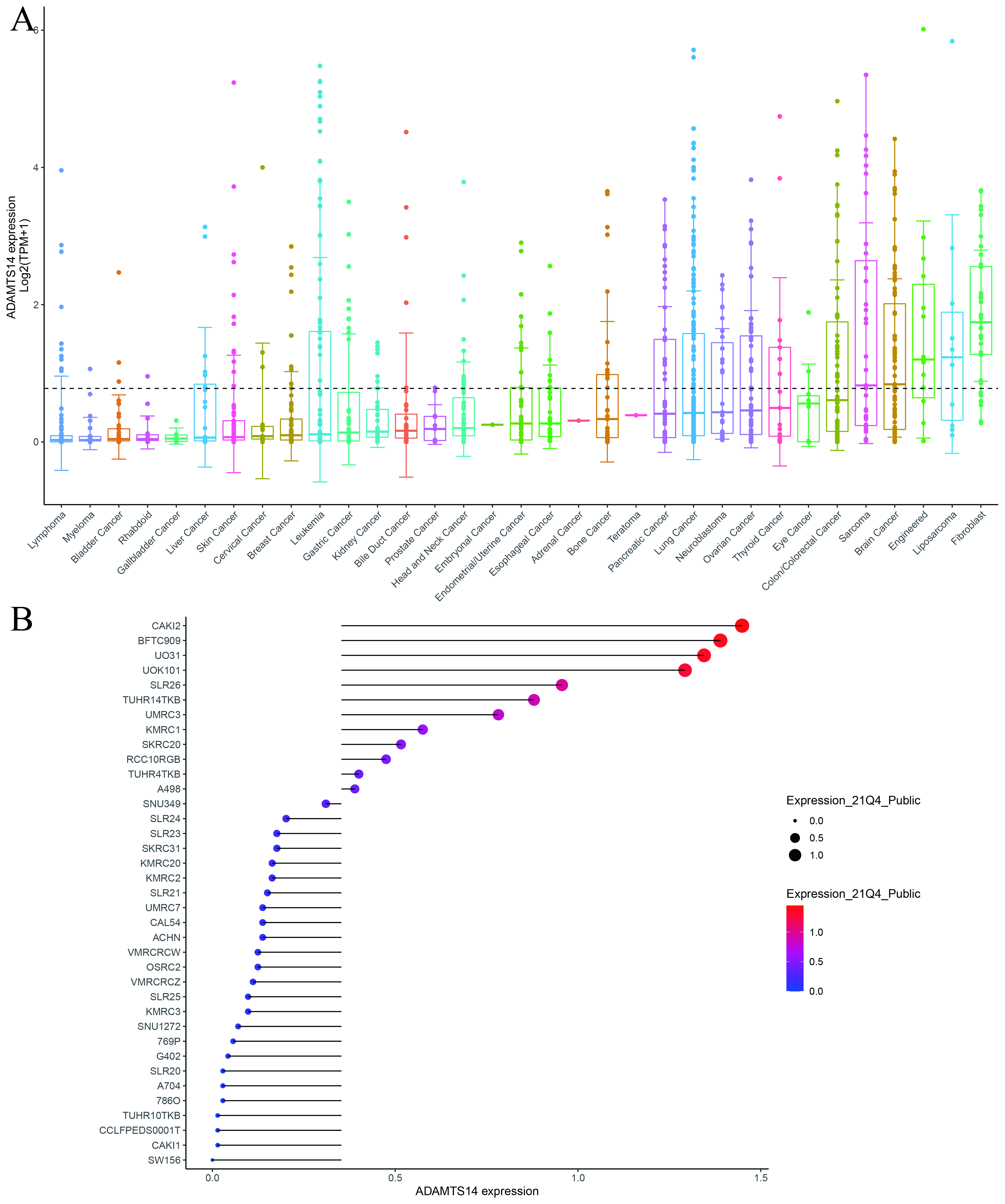

Supplement: Supplementary Figure 2 — The expression patterns of ADAMTS14 in cell lines from CCLE database; (A) Various cancer cell lines; (B) Renal cancer cell lines; [file Image_2.tif]

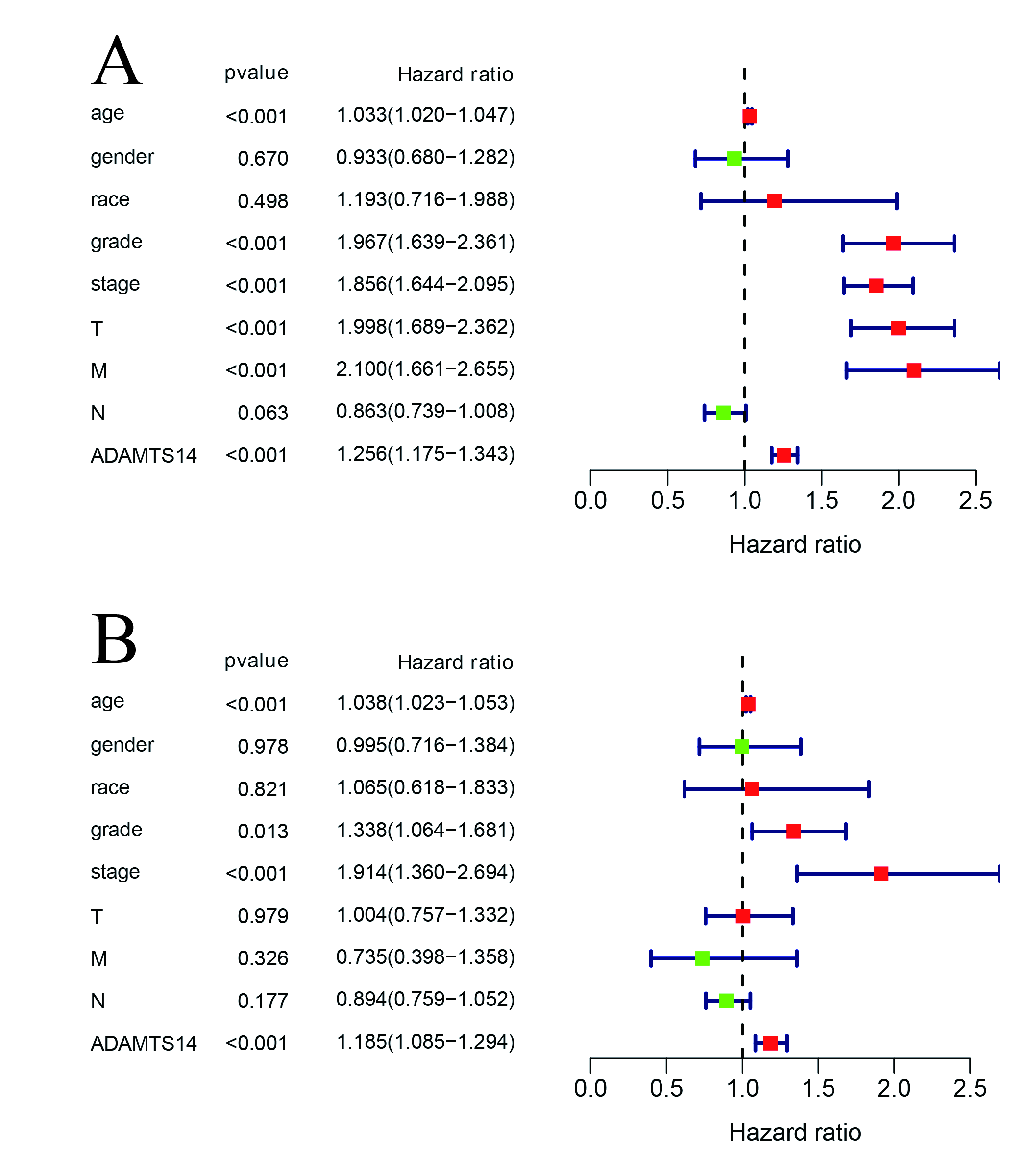

Supplement: Supplementary Figure 3 — ADAMTS14 could serve as an independent prognostic factor for ccRCC in TCGA dataset; (A) Univariate cox regression analysis; (B) Multivariate cox regression analysis; [file Image_3.tif]

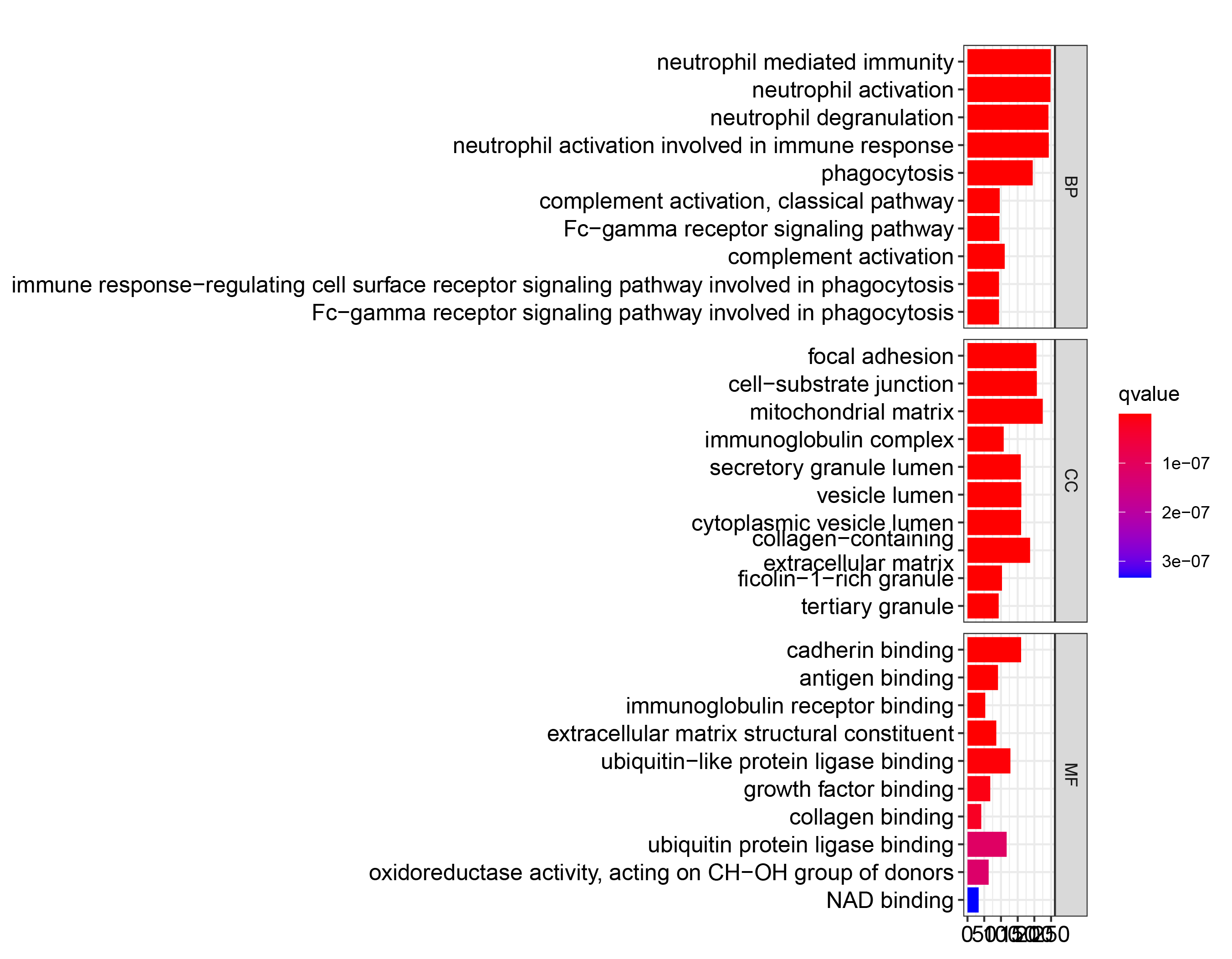

Supplement: Supplementary Figure 4 — The Gene Ontology (GO) analysis of ADAMTS14 in ccRCC patients in TCGA dataset. [file Image_4.tif]

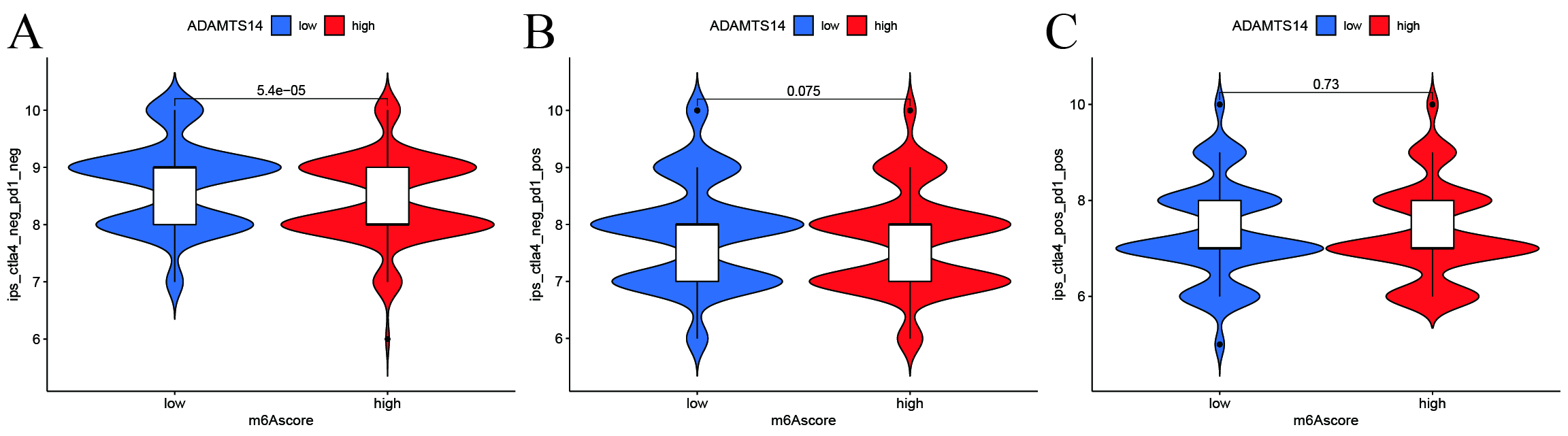

Supplement: Supplementary Figure 5 — Prediction of ADAMTS14 related immune responses of immunotherapy in ccRCC patients by TCIA dataset; (A) Distribution of ADAMTS14 expression in scores of CTLA4 negative and PD1 negative; (B) Distribution of ADAMTS14 expression in scores of CTLA4 negative and PD1 positive; (C) Distribution of ADAMTS14 expression in scores of CTLA4 positive and PD1 positive. [file Image_5.tif]
