# Supplementary material for: Survival Prognosis, Tumor Immune Landscape, and Immune Responses of ADAMTS14 in Clear Cell Renal Cell Carcinoma and Its Potential Mechanisms
Source: Front Immunol. 2022 Apr 29;13:790608. doi: 10.3389/fimmu.2022.790608 (PMC9099013; doi:10.3389/fimmu.2022.790608)
Supplement: Supplementary file 6 [file Table_1.docx]

**Table S1**: Clinical characteristics of TCGA ccRCC dataset.

| Clinical characteristics |  | Total | Proportion |
| --- | --- | --- | --- |
|  |  | （539） | （%） |
| Age at diagnosis | ≤65 | 352 | 65.3 |
|  | >65 | 185 | 34.3 |
|  | Not report | 2 | 0.4 |
| Gender | Male | 346 | 64.2 |
|  | Female | 191 | 35.4 |
|  | Not report | 2 | 0.4 |
| T | T1 | 275 | 51.0 |
|  | T2 | 69 | 12.8 |
|  | T3 | 182 | 33.8 |
|  | T4 | 11 | 2.0 |
|  | Not report | 2 | 0.4 |
| M | M0 | 426 | 79.0 |
|  | M1 | 79 | 14.7 |
|  | Not report | 34 | 6.3 |
| N | N0 | 240 | 44.5 |
|  | N1 | 17 | 3.2 |
|  | Not report | 282 | 52.3 |
| Race | ASIAN | 8 | 1.5 |
|  | BLACK OR AFRICAN AMERICAN | 56 | 10.4 |
|  | WHITE | 466 | 86.4 |
|  | Not report | 9 | 1.7 |
| Grade | G1 | 14 | 2.6 |
|  | G2 | 230 | 42.7 |
|  | G3 | 207 | 38.4 |
|  | G4 | 78 | 14.5 |
|  | Not report | 10 | 1.8 |
| Stage | Stage Ⅰ | 269 | 49.9 |
|  | Stage Ⅱ | 57 | 10.6 |
|  | Stage Ⅲ | 125 | 23.2 |
|  | Stage Ⅳ | 83 | 15.4 |
|  | Not report | 5 | 0.9 |
